# Metatranscriptomics Reveals the Functions and Enzyme Profiles of the Microbial Community in Chinese Nong-Flavor Liquor Starter

**DOI:** 10.3389/fmicb.2017.01747

**Published:** 2017-09-12

**Authors:** Yuhong Huang, Zhuolin Yi, Yanling Jin, Mengjun Huang, Kaize He, Dayu Liu, Huibo Luo, Dong Zhao, Hui He, Yang Fang, Hai Zhao

**Affiliations:** ^1^Meat-Processing Application Key Laboratory of Sichuan Province, College of Pharmacy and Biological Engineering, Chengdu University Chengdu, China; ^2^Environmental Microbiology Key Laboratory of Sichuan Province, Chengdu Institute of Biology, Chinese Academy of Sciences Chengdu, China; ^3^Key Laboratory of Environmental and Applied Microbiology, Chinese Academy of Sciences Chengdu, China; ^4^Liquor Making Bio-Technology and Application of Key Laboratory of Sichuan Province, Bioengineering College, Sichuan University of Science and Engineering Zigong, China; ^5^Wuliangye Group Yibin, China; ^6^Department of Liquor Making Engineering, Moutai College Renhuai, China

**Keywords:** ethanol fermentation, flavor generation, Chinese liquor starter, metatranscriptome, Chinese Nong-flavor liquor, saccharification

## Abstract

Chinese liquor is one of the world's best-known distilled spirits and is the largest spirit category by sales. The unique and traditional solid-state fermentation technology used to produce Chinese liquor has been in continuous use for several thousand years. The diverse and dynamic microbial community in a liquor starter is the main contributor to liquor brewing. However, little is known about the ecological distribution and functional importance of these community members. In this study, metatranscriptomics was used to comprehensively explore the active microbial community members and key transcripts with significant functions in the liquor starter production process. Fungi were found to be the most abundant and active community members. A total of 932 carbohydrate-active enzymes, including highly expressed auxiliary activity family 9 and 10 proteins, were identified at 62°C under aerobic conditions. Some potential thermostable enzymes were identified at 50, 62, and 25°C (mature stage). Increased content and overexpressed key enzymes involved in glycolysis and starch, pyruvate and ethanol metabolism were detected at 50 and 62°C. The key enzymes of the citrate cycle were up-regulated at 62°C, and their abundant derivatives are crucial for flavor generation. Here, the metabolism and functional enzymes of the active microbial communities in NF liquor starter were studied, which could pave the way to initiate improvements in liquor quality and to discover microbes that produce novel enzymes or high-value added products.

## Introduction

Chinese liquor is one of the world's four best-known distilled spirits. It accounts for more than one-third of all spirits consumed (Sweeney, 2013) and is the largest spirit category by sales in the world (Molon, 2013). The unique and traditional Chinese solid-state simultaneous saccharification and fermentation (SSF) and liquor brewing technologies have been in continuous use for several thousand years (Xiao et al., 2011; Yao et al., 2015; Xu et al., 2017). Nong-flavor (NF) liquor accounts for more than 70% of the Chinese liquor market. The main fermentation process includes two stages: liquor starter production and alcohol fermentation. The production process of NF liquor starter, which is aerobically produced, usually includes approximately 1 month of spontaneous incubation in a fermentation room and 3 months of drying in a storage room to mature (Zheng et al., 2011; Chen et al., 2014). NF liquor starters use wheat as feedstock, and the wetted wheat is shaped into bricks, each weighing approximately 1.5–4.5 kg (Zheng et al., 2011; Zheng and Han, 2016). As NF liquor starter is characterized by a moderately high temperature (62°C), the liquor starter fermenting bricks must be maintained at a moderately high temperature for 8 days in the fermentation room. For the alcohol fermentation process, first, raw sorghum, wheat, corn, rice, and sticky rice are crushed, steamed, cooled, and mixed evenly with liquor starter. Then, the mixture undergoes a solid-state SSF process for approximately 40–45 days in a pit. Finally, the fermented mixture is distilled to produce the liquor (Tao et al., 2014; Yan et al., 2015). The fermentation processes that occur during SSF are mainly attributed to the metabolism and interactions of the microorganisms from the liquor starter, Zaopei and pit mud (Chen et al., 2014). Liquor starter is the most important and essential component for liquor fermentation. During the production of liquor starter, no microorganisms are intentionally inoculated; thus, most of the microbes are enriched from naturally occurring ecosystems, such as feedstock, water, air and the working environment, with high balance and stability. These Chinese NF liquor starter microbial communities have evolved for more than several thousand years and have greatly influenced liquor properties, such as their distinctive flavor and taste.

As the NF liquor starter production process is subjected to extremely severe conditions (50–62°C), the special microbial community enriched in the liquor starter may produce efficient and diverse thermophilic carbohydrate-degrading enzymes. Recently, great efforts have been made to discover novel thermophilic lignocellulases with excellent performance, including high activity and marked stability (McClendon et al., 2012; Balasubramanian and Simões, 2014). The carbohydrate-degrading enzymes from the liquor starter under aerobic and thermophilic conditions are different from those in termite and other herbivore-associated gut communities, which are dominated by anaerobic bacteria. Thus, enzymes from liquor starter may have great potential for industrial applications because lignocellulose decomposition has mainly been demonstrated under aerobic conditions (Robinson et al., 2001). Therefore, global and comprehensive technologies are needed to retrieve multiple thermophilic and synergistic carbohydrate-degrading enzymes from the NF liquor starter system. Elucidating the saccharification capability of liquor starters and identifying other attractive enzymes for industrial applications would also be of great value.

The microbial community of liquor starters has been studied using culture-dependent and denaturing gradient gel electrophoresis methods (Zheng et al., 2012; Yan et al., 2013; Chen et al., 2014; Zhang L. et al., 2014; Wang and Xu, 2015) as well as pyrosequencing techniques (Li et al., 2013; Zhang X. et al., 2014; Wang et al., 2017). Moreover, Huang et al. (unpublished data) compared the dominant microbial community of Jiang-flavor (JF) and NF liquor starters and provided a more complete picture of the microbial composition in liquor starters. These studies have increased the understanding of the microbial community structure of liquor starters. However, not all of these methods are ideal for assessing community functions, and little is known concerning the active microbial community compositions and their metabolic functions in liquor starter. Metatranscriptomics, the direct analysis of mRNA from environmental samples, offers a powerful tool to study the active microbial community composition as well as their active genes and changes in transcriptional regulation when microbes respond to temporal variation (Mitra et al., 2011). Although, several studies have demonstrated the great advantage of metatranscriptomic technology (Bashir et al., 2013; Sanders et al., 2013; Wu et al., 2013), high-quality RNA from complex and difficult environmental samples severely challenges metatranscriptomics projects. The high content of starch and other polysaccharides, the complex fermentation products and the strong colored biomass during fermentation make RNA extraction of the microbial community in liquor starter difficult.

In the present study, we first successfully extracted total RNA from complex liquor starter samples and then applied high-throughput metatranscriptomic technology to globally, comprehensively and functionally analyze the actual microbial composition and metabolic characteristics of the most widely consumed NF liquor starter during the production process. The efforts of this study provide the first step in understanding the metabolism and function of the active microbial communities in liquor starters and pave the way toward the optimization of liquor production, improvement of liquor quality and discovery of microbes that produce valuable and novel enzymes with great potential for industrial applications.

## Materials and methods

### Sample collection

NF liquor starter was sampled from a fermentation workshop of the Yibin Hongloumeng Distillery Group Co., Ltd. in Yibing, Sichuan, China, in July 2013. The liquor starter was sampled at different time points. The samples were harvested from three locations in the same liquor starter fermentation room at each time point. N1 was collected at the beginning of liquor starter fermentation (30°C); N2 was collected after 3 days of liquor starter fermentation (50°C); N3 was collected after 9 days of liquor starter fermentation (62°C); and N4 was collected from the mature liquor starter after fermentation for 20 days (25°C). The samples were frozen in liquid nitrogen when they were harvested in the fermentation workshop and were then immediately transferred to 50-ml RNase free Corning CentriStar™ centrifuge tubes (430828, Corning, NY, USA) and kept on dry ice. Finally, all the samples were transferred to the Chengdu Biology Institute at the Chinese Academy of Sciences on the day of sampling and stored in a −80°C freezer until analyses. The liquor starter samples for enzyme analysis were prepared as follows: 5 g of liquor starter was suspended in 20 ml of 0.1% (v/v) Tween 80 solution and transferred to the Chengdu Biology Institute, Chinese Academy of Sciences at room temperature.

### Enzyme profile of the NF liquor starter

After the liquor starter in the Tween 80 solution was transferred to the laboratory, the samples were incubated at 25°C with shaking at 100 rpm overnight. The enzyme profile of the supernatant was investigated using insoluble chromogenic AZurine Cross-Linked (AZCL) polysaccharides according to the manufacturer's protocol (Megazyme, Ireland). After incubation at 35, 45, or 55°C for 22 h, the diameter of the blue haloes were measured and recorded in millimeters.

### RNA extraction

Total RNA was extracted from liquor starter according to a previously reported method (Kumar et al., 2011) with some modifications. Briefly, 1 g of liquor starter was homogenized into fine powder in a precooled mortar with liquid nitrogen. Next, 4 ml of a hot (80°C) borate buffer [200 mM sodium borate (pH 9.0), 30 mM ethyleneglycotetraacetic acid (EGTA), 1% (w/v) sodium dodecyl sulfate (SDS), 2% (w/v) polyvinylpyrrolidone (PVP), and 0.5% (v/v) Nonidet-40 (NP-40) combined in 0.1% diethyl pyrocarbonate (DEPC)-treated water and then autoclaved; after cooling and just before use, 10 mM β-mercaptoethanol and 0.03% (v/v) RNase inhibitor were added] and 280 μl of proteinase K (20 mg/ml) were added, and the mixture was incubated at 80°C for 2 min. The lysate was centrifuged for 10 min at 5,000 × g. The supernatant was mixed thoroughly with an equal volume of 70% ethanol by shaking vigorously. The sample was applied to an RNeasy midi column and centrifuged for 5 min at 5,000 × g; this step was repeated for the residual sample. The sample was cleaned following the RNeasy Midi Kit protocol (Qiagen, 75142) and treated with DNase I (Fermentas, USA) according to the manufacturer's protocol. The purity, concentration and RNA integrity number (RIN) were measured using an Agilent 2100 Bioanalyzer. Qualified total RNA was submitted to the Beijing Genomics Institute (BGI)-Shenzhen, China, for RNA sequencing.

### cDNA illumina library construction, RNA sequencing and *de novo* assembly

More than 20 μg of qualified total RNA from each sample (N1, N2, N3, and N4) was used for RNA sequencing using the HiSeq™ 2000 platform. For eukaryotes, poly (A) mRNA was purified using poly-T oligo-attached magnetic beads. For prokaryotes, rRNA was removed before subsequent library construction steps. The mRNA was mixed with fragmentation buffer and then fragmented. Fragmented mRNAs were synthesized into first-strand cDNA using reverse transcriptase and random primers. This step was followed by second-strand cDNA synthesis. Short fragments were purified and resolved with EB buffer for end reparation and poly (A)-tailing. Thereafter, the short fragments were connected with sequencing adapters, and then 200-bp cDNA fragments were purified for further template enrichment by PCR. The validated 200-bp fragment cDNA libraries were submitted for paired-end (PE) RNA sequencing using the HiSeq™ 2000 platform. Known bacterial, fungal and archaeal sequences were extracted from the National Center for Biotechnology Information (NCBI) Nucleotide (NT) database using in-house scripts, and the filtered reads were mapped to these sequences using the SOAP aligner (version 2.21) (Li et al., 2009). Next, the transcriptome data were assembled *de novo* using Trinity (http://trinityrnaseq.sourceforge.net/; Grabherr et al., 2011). The raw and assembled sequencing data have been deposited in the DDBJ/EMBL/GenBank database under the accession numbers SRR5384077 and GFMA00000000, respectively.

### Functional annotation and cluster analysis

The software TransDecoder (http://sourceforge.net/projects/transdecoder/) was used to predict open reading frames (ORFs) based on the assembly results. The predicted amino acid sequences were aligned to diverse databases through BLAST (version 2.2.23), and related information was extracted and summarized using custom scripts. Gene Ontology (GO) classification (Ashburner et al., 2000) was achieved using WEGO (http://wego.genomics.org.cn/cgi-bin/wego/index.pl) (Ye et al., 2006). Enzyme codes were extracted, and the Kyoto Encyclopedia of Genes and Genomes (KEGG) pathways were retrieved from the KEGG web server (Kanehisa, 1997; Kanehisa et al., 2004, 2006). Carbohydrate-active enzymes were annotated according to the Carbohydrate-Active enZymes database (CAZy) (version: 2011-9-20) (Cantarel et al., 2009). The evolutionary genealogy of genes was extracted from Non-supervised Orthologous Groups (eggNOG) (version 3.0) (Powell et al., 2012). KEGG, GO CAZy and eggNOG function cluster analyses were conducted using custom scripts. A heat map was constructed with the R package (Ihaka and Gentleman, 1996) using custom scripts.

### Expression profiling and differential expression identification

To investigate the expression level of each unigene in the different samples, all the predicted ORFs were removed for redundancy using cd-hit (Version 4.6.1, http://weizhong-lab.ucsd.edu/cdhit_suite/cgi-bin/index.cgi). Unigene expression was calculated according to the fragments per kilobase of transcripts per mapped million fragments method (FPKM) (Ali et al., 2008). The *P*-values and log2-fold-changes (log_2_FCs) were calculated, and then the significantly differentially expressed transcripts (DETs) between the two samples were identified using *p* ≤ 0.05 and log_2_FC ≥ 1. Because thousands of hypothesis tests were performed using the transcriptome data, a suitable *p*-value for an individual test is not sufficient to guarantee a low rate of false discovery. Thus, multiple testing corrections for each individual hypothesis were performed to guarantee an overall low false discovery rate. The false discovery rate (FDR) control is a statistical method used in multiple hypothesis testing to correct for the *p*-value as described previously (Benjamini and Yekutieli, 2001). When the FDR was obtained, the FPKM ratio of the two samples was used at the same time. In this analysis, the values were identified as follows: FDR ≤ 0.001 and FPKM ratio ≥ 2.

## Results

### Metatranscriptome sequencing and *de novo* assembly of the NF liquor starter samples

After sequencing, we obtained 21.789 Gbp of data in total (Table S1). The raw reads were cleaned, pooled and assembled *de novo*. The assembly metrics can be found in Table S2. The maximum contig lengths were 13,340, 17,819, 12,496, and 12,933 bp, and the N_50_ lengths were 404, 538, 1,084, and 1,105 bp for N1, N2, N3, and N4, respectively. The coding DNA and protein sequences were predicted and translated based on the assembled transcripts. Scanning the ORFs of all the contigs identified 25387, 58884, 56927, and 28618 ORFs with lengths longer than 600 bp for N1, N2, N3, and N4, respectively (Table S3).

### Functional profiling and characterization of the NF liquor starter metatranscriptome

The high-quality sequences were aligned to the bacterial and fungal sequences in the NT database. The composition of active species is presented in Table 1. The identified active fungal community was more prevalent than the bacterial community during the entire liquor starter production process as more active mRNA can be detected. The fungal component even increased up to 66.22% for sample N4. However, the bacterial component increased to the highest value of 15.30% for sample N3 and dropped to 12.33% for sample N4.

**Table 1:** Species information for the liquor starter samples after blasting the cDNA sequencing reads against the bacterial and fungal databases.

**Sample ID**	**Database**	**Rate (%)**
N1	Bacteria	1.10
N1	Fungi	15.43
N2	Bacteria	7.68
N2	Fungi	48.35
N3	Bacteria	15.30
N3	Fungi	45.05
N4	Bacteria	12.33
N4	Fungi	66.22

GO, CAZy, eggNOG, and KEGG annotation combined with BLASTX was performed to profile the active genes and related pathways in the NF liquor starter. A total of 29418 unigenes from all the NF samples were annotated with the GO database, accounting for 17.32% of all the unigenes. The annotation was mainly clustered into three general sections: biological processes, cellular components and molecular functions (Figure S1). Primary metabolic processes and catalytic activities were the most enriched GO terms in the biological processes and molecular functions sections, respectively. This result was further confirmed by KEGG analysis. For the NF liquor starter samples N1, N2, N3, and N4, 5047, 16419, 17034 and 8662 unigenes were found in 254, 264, 260, and 250 reference pathways, accounting for 19.88%, 27.88, 29.92, and 30.27% of the total unigenes, respectively.

**Figure 1:**
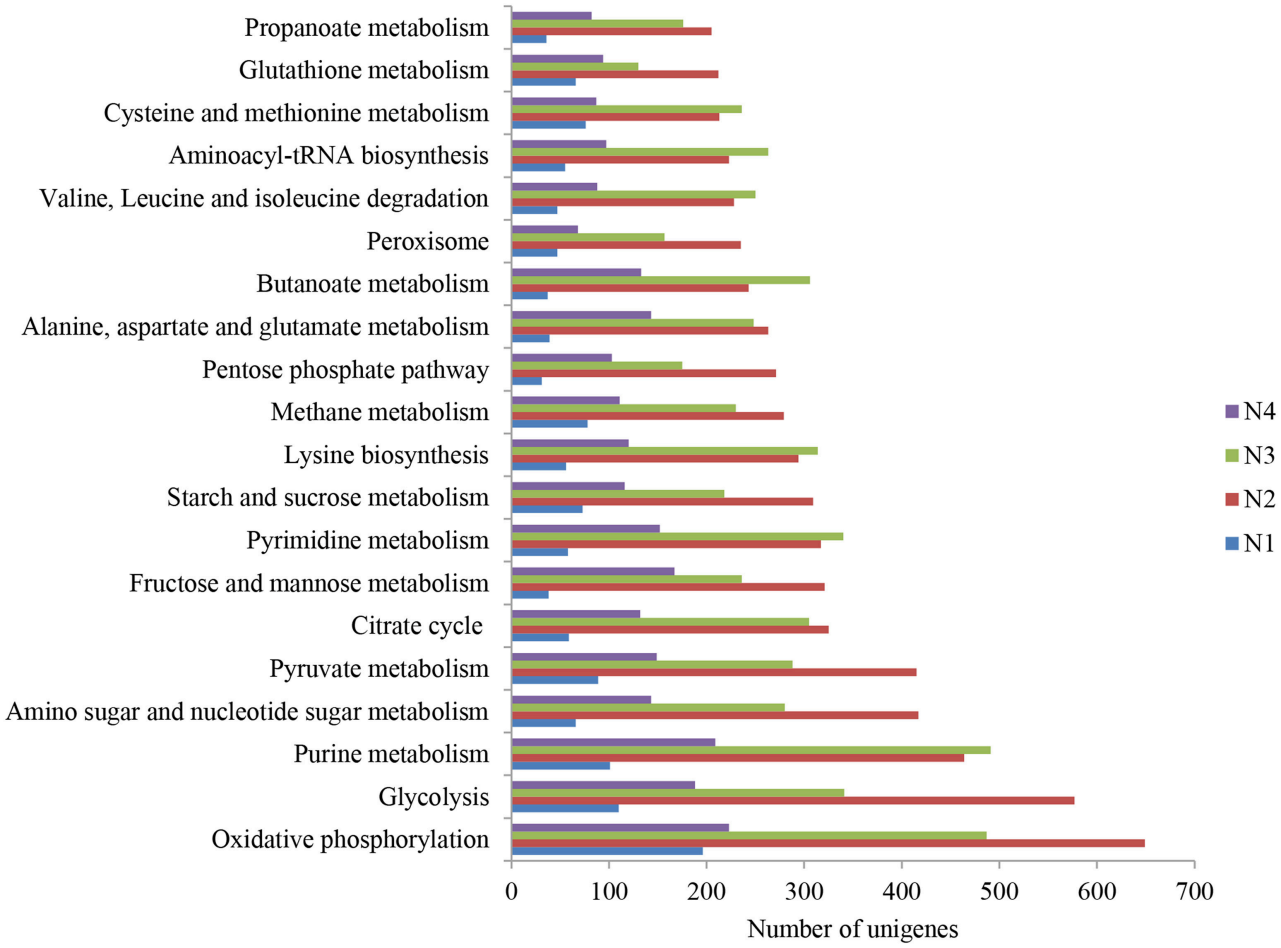
The 20 most abundant KEGG pathways in the metatranscriptome of Nong-flavor liquor starter samples (N1, N2, N3, and N4). N1 was sampled at the beginning of liquor starter production, N2 was sampled after 3 days of liquor starter fermentation, N3 was sampled after 9 days of liquor starter fermentation, and N4 was the mature liquor starter. The temperatures of N1, N2, N3, and N4 were 30, 50, 62 and 25°C, respectively.

The 20 most abundant KEGG pathways of the four samples are shown in Figure 1. Notably, oxidative phosphorylation was ranked as the first most abundant pathway for sample N2. Among the 20 pathways, glycolysis, pyruvate metabolism, the citrate cycle, fructose and mannose metabolism, starch and sucrose metabolism and the pentose phosphate pathway were highly represented, particularly in samples N2 and N3. Additionally, butanoate metabolism and propanoate metabolism were found among the 20 pathways. Metabolism of 10 amino acids, i.e., lysine, alanine, aspartate, glutamate, valine, leucine, isoleucine, cysteine, methionine and glutathione, were also ranked in the top 20 and were dominant in samples N2 and N3.

**Figure 2:**
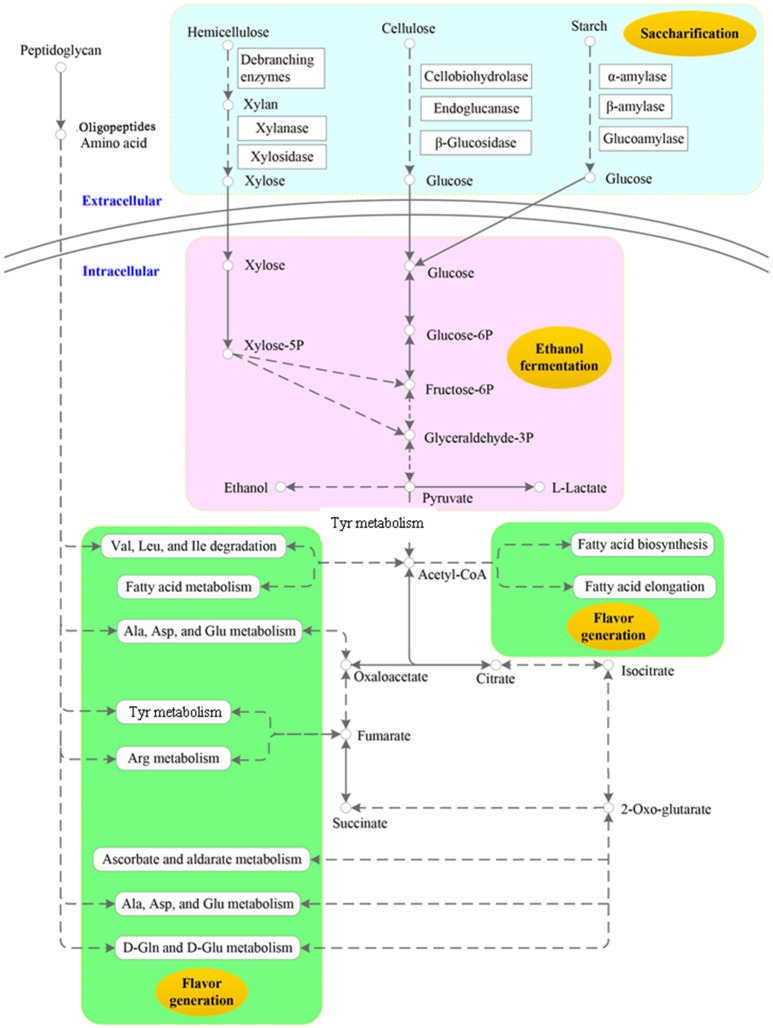
Key metabolic profiles of scarification, ethanol fermentation and flavor generation in the Nong-flavor liquor starter. In this schematic summary, extracellular and intracellular reactions are separated by the cell membrane, but these reactions are not restricted to one cell. The light-blue color indicates the scarification profile. The pink color indicates the ethanol fermentation profile, and the green color indicates that the derivatives may be related to flavor generation.

The specific pathways related to saccharification, ethanol fermentation and flavor generation in the NF liquor starter metatranscriptome are schematically presented in Figure 2. First, polymers such as cellulose, hemicellulose, starch and protein are converted to monomers by diverse carbohydrate-active enzymes and proteases with high activity. Next, the monomers are taken up and further utilized by the microbial community. The products and intermediates of primary metabolism, such as glycolysis and the citrate cycle, as well as by-products of metabolism, mainly contribute to ethanol production and flavor development.

### Identification of carbohydrate-active enzymes

In the present study, we analyzed the enzyme profiles of the NF liquor starter samples at different reaction temperatures (35, 45, and 55°C). Low enzyme activity was detected in the initial sample (N1) (Table 2). However, a broad spectrum of carbohydrate-active enzymes was detected in sample N2. More importantly, endo-β-1,3-1,4-glucanase, endo-1,3-β-D-glucanase, α-amylase, endo-1,5-α-L-arabinanase and endo-protease thermophilic activity was obviously present with higher activity at higher temperatures. Furthermore, endo-β-1,3-1,4-glucanase, rhamnogalacturonanase and endo-protease thermostable activity was clearly present in the N3 sample. The mature liquor starter sample N4 also had a broad spectrum of enzymatic activity.

**Table 2:** Carbohydrate-active enzyme analysis for Nong-flavor liquor starter using insoluble chromogenic AZurine Cross-Linked (AZCL) polysaccharides.

**Substrate**	**Enzyme**	**Diameter (mm)**			
		**N1**	**N2**	**N3**	**N4**
		**35/45/55°C**	**35/45/55°C**	**35/45/55°C**	**35/45/55°C**
AZCL-beta-glucan	Cellulase (endo-β-1,3-1,4-glucanase)	3/3/3	17.5/20/23	7/8/13	13/16/22
AZCL-galactan	endo-1,4- β -D-galactanase		0.5/0.5/0	6/9/2	12/12/7
AZCL-curdlan	endo-1,3- β -D-glucanase	4/4/4	10/15/18		4/9.5/7
AZCL-amylose	α-amylase	2/6/6	9.5/17/17	3/4/4	14.5/15/16
AZCL-collagen	endo-proteases				
AZCL-debranched arabinan	endo-1,5-α-L-arabinanase	0.5/0.5/0.5	0/7.5/8	7/7/6	10/8/10
AZCL-galactomannan	endo-1,4- β -D-mannanase	5.5/6/5	8/14/13	11/3/0.5	6.5/8/8
AZCL-xyloglucan	endo-β-1,4-xyloglucanase		5.5/5/7		
AZCL-xylan	endo-1,4- β -D-xylanase				13/16/18
AZCL-he-cellulose	cellulase (endo-β-1,4-glucanase)				
AZCL-pullan	microbial pullulanase				
AZCL-chitosan	Chitosanase				
AZCL-dextran	endo-1,6-α-D-glucanase				
AZCL-rhmnogalacturonan I	Rhamnogalacturonanase		14/3/4	7/8/11	3/15/17
AZCL-casein	endo-proteases		7/14/14	0/6/6	6/12/12.5
AZCL-arabinoxylan	endo-1,4- β -D-xylanase			4/3/0	11/21.5/22

**Figure 3:**
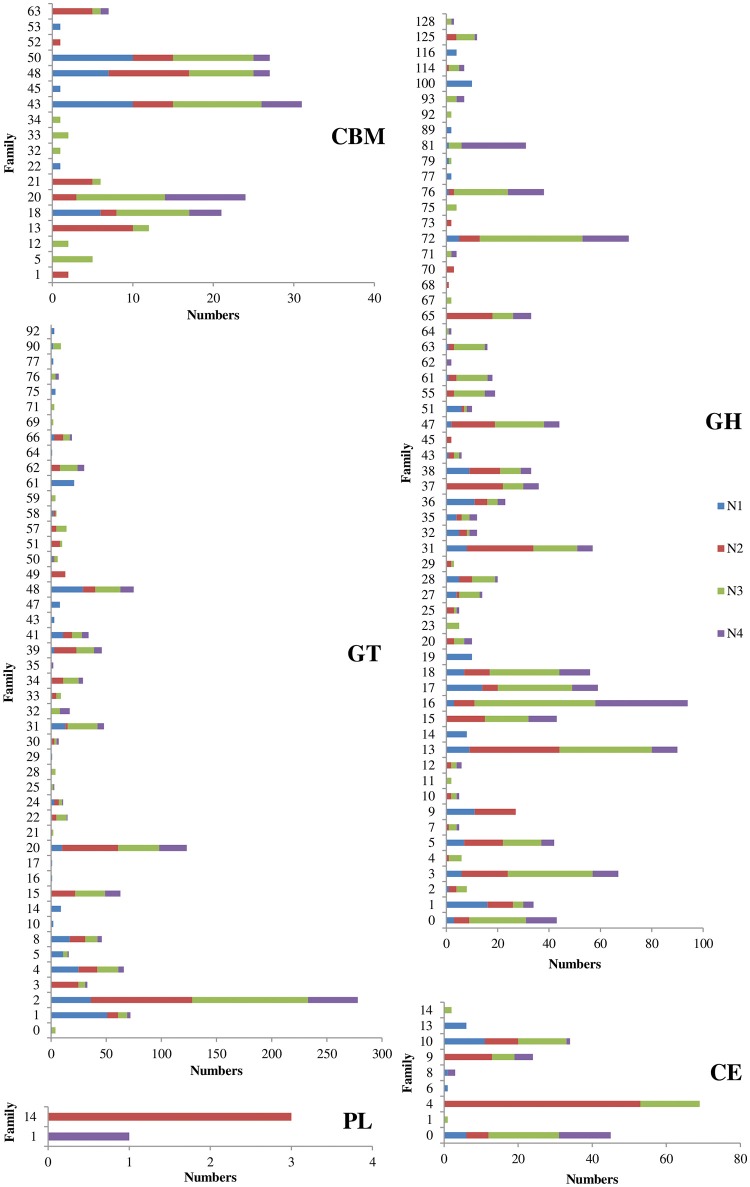
Matched numbers of carbohydrate-active enzymes from the Nong-flavor liquor starter samples. CBM, Carbohydrate-Binding Module; GT, Glycosyl Transferase; PL, Polysaccharide Lyase; GH, Glycoside Hydrolase; and CE, Carbohydrate Esterase. N1 was sampled at the beginning of liquor starter production, N2 was sampled after 3 days of liquor starter fermentation, N3 was sampled after 9 days of liquor starter fermentation, and N4 was the mature liquor starter. The temperatures of N1, N2, N3, and N4 were 30, 50, 62, and 25°C, respectively.

The metatranscriptome data were annotated to further identify carbohydrate-active enzymes at the molecular level. The profiles of the carbohydrate-active enzymes varied among the four samples (Figure 3). The N3 sample had the highest number of carbohydrate-active enzymes, including 478 glycoside hydrolases, 397 glycosyl transferases, 57 carbohydrate esterases and 64 carbohydrate-binding modules, followed by the N2 sample (Figure 3 and Table S4). The most highly expressed glycoside hydrolases in the NF liquor starter were mainly classified as cellulases (GH5, GH7, GH9 and GH45), endo-hemicellulases (GH10, GH11, GH12 and GH28), cell wall extension enzymes (GH16, GH17 and GH81), cell wall debranching enzymes (GH51 and GH67) and oligosaccharide-degrading enzymes (GH1, GH2, GH3, GH29, GH35, GH38, and GH43).

In this study, the most abundant and diverse auxiliary activity 9 (AA9) genes (up to 12 unique encoding genes) were found in the N3 sample (Table 3). The AA9 genes were mainly from *Thermoascus aurantiacus, Trichocomaceae* and *Emericella nidulans*. Only one AA10 protein from *Bacillales* was found in the N3 sample. All of these AA9 and AA10 proteins secreted by thermophilic fungi and bacteria were first identified in the NF liquor starter with low identity (41–75%) to the reported protein sequences. Additionally, the phylogenetic tree (Figure S2) showed that the AA9 proteins are diverse in sequence.

**Table 3:** Inventory of putative AA9 and AA10 proteins in Nong-flavor liquor starter at different time periods.

**Sample**	**Gene_id**	**Identity (%)**	**Family**	**Species information**
N1	N1_25028	59.56	AA9	*Pyrenophora*
N2	N2_38569	71.26	AA9	*Thermoascus aurantiacus*
	N2_47469	72.87	AA9	*Trichocomaceae*
	N2_47471	75.18	AA9	*Eurotiomycetidae*
N3	N3_22651	71.26	AA9	*Thermoascus aurantiacus*
	N3_25796	72.87	AA9	*Trichocomaceae*
	N3_25797	73.39	AA9	*Trichocomaceae*
	N3_27422	65.71	AA9	Unknown
	N3_48067	75.81	AA9	*Thermoascus aurantiacus*
	N3_49513	40.82	AA9	*Trichocomaceae*
	N3_53678	42.57	AA9	*Trichocomaceae*
	N3_31319	–	AA9	*Paracoccidioides brasiliensis*
	N3_6630	58.86	AA9	*Emericella nidulans*
	N3_7220	41.74	AA9	*Trichocomaceae*
	N3_8741	72.87	AA9	*Trichocomaceae*
	N3_14765	43.21	AA9	*Emericella nidulans*
	N3_43070	–	AA10	Bacteria
	N3_51026	73.55	AA10	*Bacillales*
N4	N4_12386	61.65	AA9	*Trichocomaceae*
	N4_26828	66.52	AA9	*Emericella nidulans*

### Starch metabolism, glycolysis, ethanol metabolism, and pyruvate metabolism

**Figure 4:**
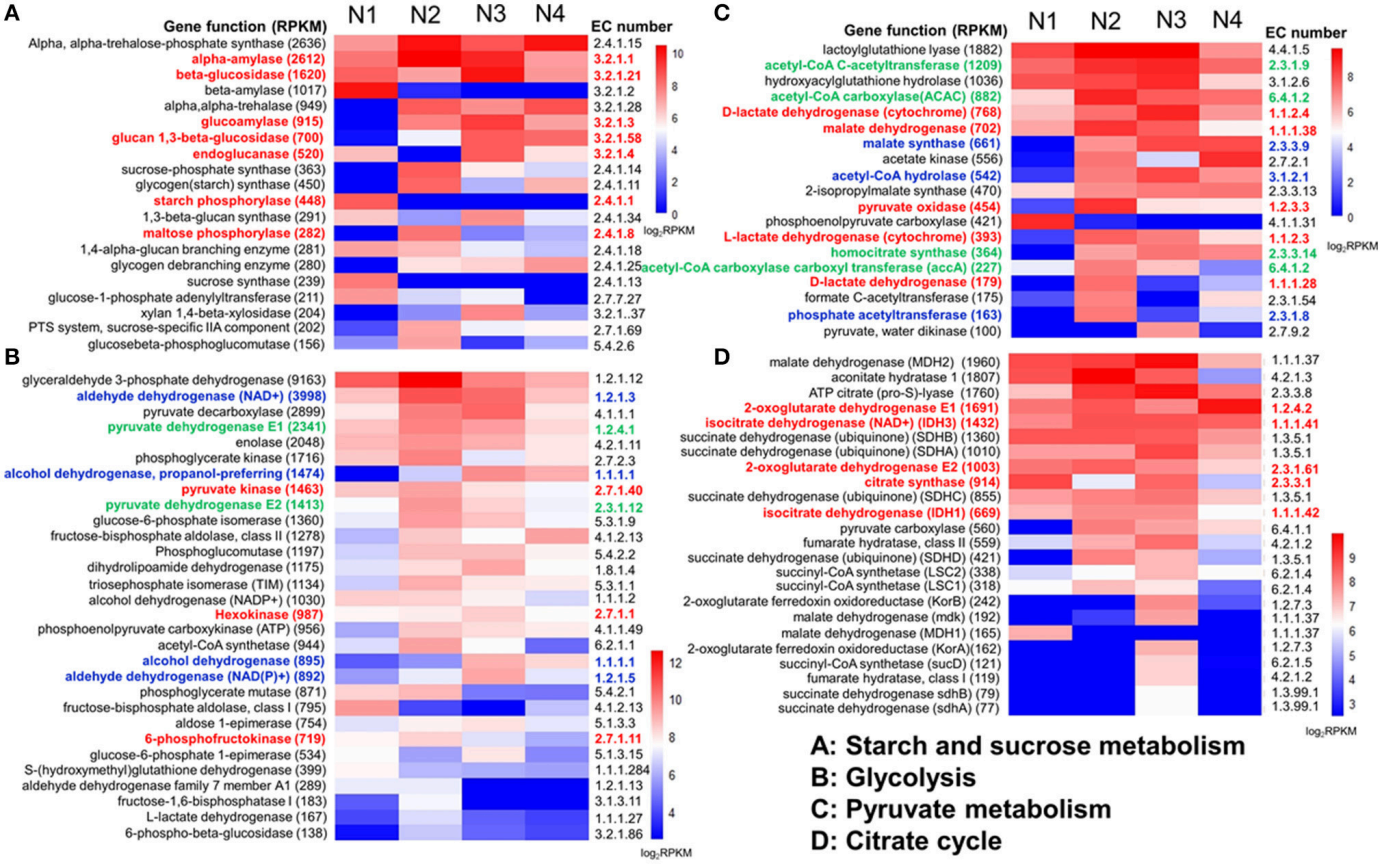
Genes related to carbohydrate and energy metabolism were relatively highly expressed in the liquor starter samples (N1, N2, N3, and N4). Four abundant carbohydrate and energy metabolisms were analyzed here, i.e., starch and sucrose metabolism **(A)**, glycolysis **(B)**, pyruvate metabolism **(C)**, and the citrate cycle **(D)**. For each metabolism, relatively high gene expression levels were presented by function, EC number and total RPKM. Relative expression (log_2_RPKM)) is shown between the high (red) and low (blue) expression levels. The key enzymes are highlighted with color.

Enzymes related to starch metabolism were analyzed among the four samples (Figure 4A). Most of the enzymes were more abundant in the N2 and N3 samples, especially the important enzymes related to the conversion of starch to glucose, such as α-amylase, starch phosphorylase, maltose phosphorylase, β-glucosidase, glucoamylase, glucan 1,3-β-glucosidase and endoglucanase (colored in red in Figure 4A). Moreover, α-amylase had the highest expression level, with an RPKM value of up to 1398.5 in the N2 sample.

We then comparatively analyzed the glycolysis metabolism of the NF liquor starter samples. The results indicated that there were various expression levels for the 9 enzyme-catalyzed reactions that convert hexose to pyruvate in all the samples (Figure S3). The N2 sample had the highest total numbers and expression levels of the glycolysis genes (Figure 1 and Figure 4B). Among these genes, glyceraldehyde 3-phosphate dehydrogenase had the highest expression level with an RPKM value of 6004.1. Many of the enzyme genes also exhibited comparable expression levels in sample N3 (Figure 4B). Furthermore, three key enzymes in glycolysis, hexokinase, 6-phosphofructokinase and pyruvate kinase (colored in red in Figure 4B), were significantly up-regulated, with log_2_Ratio values (N2/N1) ranging from 3.6 to 16.6 (Figure S3 and Table S5). In total, 8 hexokinases, 10 6-phosphofructokinases and 16 pyruvate kinases were up-regulated, and the up-regulated genes were mainly from *Saccharomycetales* and *Mucorales*.

Pyruvate can be converted to ethanol in conditions of insufficient oxygen, which possibly occurs inside the liquor starter brick. As shown in Figure S3, alcohol dehydrogenase, aldehyde dehydrogenase (NAD+) and aldehyde dehydrogenase (NAD(P)+) are three key enzymes in ethanol metabolism. The highest expression levels of alcohol dehydrogenase and aldehyde dehydrogenase (NAD(P)+) were in N3, and the highest expression level of aldehyde dehydrogenase (NAD+) (RPKM: 1878.7) was in N2, with a comparable level (RPKM: 1300.4) in N3 (colored in blue in Figure 4B). Furthermore, compared with the N1 sample, the alcohol dehydrogenase levels in the N2, N3, and N4 samples from different microbial community members were up-regulated. In particular, in the N2 sample, 10 types of alcohol dehydrogenases were up-regulated from diverse microorganisms, such as *Leuconostocaceae, Millerozyma farinose, Weissella thailandensis, Saccharomycetales, Dikarya, Rhizopus oryzae*, and *Lactobacillales* (Table 4). The log_2_Ratio (N2/N1) values of the alcohol dehydrogenases varied from 9.5 to 14.2. When the temperature was increased to 62°C, some aldehyde dehydrogenases (NAD+) and aldehyde dehydrogenases (NAD(P)+) were also up-regulated in the N3 sample. The RPKM value of one aldehyde dehydrogenase (NAD(P)+) produced by *Trichocomaceae* increased from 9.7 to 237.6, and the log_2_Ratio (N3/N2) value of aldehyde dehydrogenase (NAD+) produced by bacteria was the highest value of 15.6 (Table 5).

**Table 4:** Changes in alcohol dehydrogenase (EC1.1.1.1), a key enzyme in the ethanol metabolism pathway, between liquor starter samples N2 and N1.

**GeneID**	**Ko_name**	**Ko_defi**	**Ko_EC**	**N1-RPKM**	**N2-RPKM**	**log_2_ Ratio (N2/N1)**	**Up-Down-Regulation (N2/N1)**	***P*-value**	**FDR**	**Species information**
N2_19311	adh	Alcohol dehydrogenase	1.1.1.1	0.0	6.4	12.7	Up	0.1625	0.1940	*Leuconostocaceae*
N2_23390	adhP	Alcohol dehydrogenase. Propanol-preferring	1.1.1.1	0.0	6.9	12.8	Up	0.0983	0.1224	*Millerozyma farinosa*
N2_24864	adhP	Alcohol dehydrogenase. Propanol-preferring	1.1.1.1	0.0	16.0	14.0	Up	0.0021	0.0033	*Leuconostocaceae*
N2_25713	adh	Alcohol dehydrogenase	1.1.1.1	0.0	3.3	11.7	Up	0.5243	0.5758	*Leuconostocaceae*
N2_29242	adh	Alcohol dehydrogenase	1.1.1.1	0.0	18.3	14.2	Up	0.0000	0.0001	*Weissella thailandensis*
N2_29640	adhP	Alcohol dehydrogenase. Propanol-preferring	1.1.1.1	0.0	12.9	13.7	Up	0.0029	0.0046	*Saccharomycetales*
N2_700	adhP	Alcohol dehydrogenase. Propanol-preferring	1.1.1.1	0.0	0.7	9.5	Up	0.8662	0.8807	*Dikarya*
N2_38029	adh	Alcohol dehydrogenase	1.1.1.1	0.0	11.9	13.5	Up	0.0001	0.0002	*Rhizopus oryzae*
N2_38923	adhP	Alcohol dehydrogenase. Propanol-preferring	1.1.1.1	0.0	11.6	13.5	Up	0.0029	0.0046	*Saccharomycetales*
N2_15497	adhP	Alcohol dehydrogenase. Propanol-preferring	1.1.1.1	0.0	19.2	14.2	Up	0.0001	0.0001	*Lactobacillales*

**Table 5:** Changes in aldehyde dehydrogenase (NAD+) (EC1.2.1.3) and aldehyde dehydrogenase (NAD(P)+) (EC1.2.1.5), key enzymes in the ethanol metabolism pathway, in liquor starter samples N3 and N2.

**GeneID**	**Ko_defi**	**Ko_EC**	**N2-RPKM**	**N3-RPKM**	**log_2_ Ratio (N3/N2)**	**Up-Down-Regulation (N3/N2)**	***P*-value**	**FDR**	**Species information**
N3_18183	Aldehyde dehydrogenase (NAD+)	1.2.1.3	0.0	32.5	15.0	Up	4.44E-16	1.89E-15	Bacteria
N3_20191	Aldehyde dehydrogenase (NAD+)	1.2.1.3	0.0	50.5	15.6	Up	4.44E-16	1.65E-15	Bacteria
N3_20791	Aldehyde dehydrogenase (NAD+)	1.2.1.3	0.0	23.3	14.5	Up	4.44E-16	1.40E-15	*Bacillales*
N3_2443	Aldehyde dehydrogenase (NAD+)	1.2.1.3	0.0	27.0	14.7	Up	4.44E-16	1.87E-15	Bacteria
N3_19461	Aldehyde dehydrogenase (NAD(P)+)	1.2.1.5	0.0	16.7	14.0	Up	4.44E-16	1.32E-15	*Trichocomaceae*
N3_20602	Aldehyde dehydrogenase (NAD(P)+)	1.2.1.5	0.0	15.3	13.9	Up	4.44E-16	1.48E-15	*Trichocomaceae*
N3_20661	Aldehyde dehydrogenase (NAD(P)+)	1.2.1.5	0.0	41.9	15.4	Up	4.44E-16	1.40E-15	*leotiomyceta*
N3_21915	Aldehyde dehydrogenase (NAD(P)+)	1.2.1.5	9.7	237.6	4.6	Up	4.84E-14	1.29E-13	*Trichocomaceae*
N3_39954	Aldehyde dehydrogenase (NAD(P)+)	1.2.1.5	12.0	159.3	3.7	Up	0	0	*Eurotiomycetidae*
N3_16852	Aldehyde dehydrogenase (NAD(P)+)	1.2.1.5	2.2	61.0	4.8	Up	3.92E-08	7.09E-08	*Trichocomaceae*

Another important intermediate of pyruvate metabolism is acetyl-CoA, which is produced by pyruvate dehydrogenase E1 and pyruvate dehydrogenase E2 (Figures S3, S4). Pyruvate dehydrogenases E1 and E2 (colored in green in Figure 4B) both showed their highest expression levels in the N2 sample, which was followed by N3. Meanwhile, other enzymes involved in the complicated metabolism of pyruvate were also compared among the four samples. Most of them showed relatively high expression levels in the N2 and N3 samples (Figure 4C and Table S6). For the 19 most highly expressed pyruvate metabolism enzymes, 8 enzymes exhibited their highest RPKM values in the N2 sample, and 7 enzymes exhibited their highest RPKM values in N3. Malate dehydrogenase, D-lactate dehydrogenase/D-lactate dehydrogenase (cytochrome), L-lactate dehydrogenase and pyruvate oxidase (colored in red in Figure 4C) are responsible for reversibly converting pyruvate to L-malate, D- lactate, L-lactate and acetyl phosphate, respectively. Meanwhile, phosphate acetyltransferase, malate synthase and acetyl-CoA hydrolase (colored in blue in Figure 4C) are responsible for irreversibly converting acetyl-CoA to acetyl phosphate, L-malate and acetate, respectively. More importantly, acetyl-CoA carboxylase (ACAC/accA), acetyl-CoA C-acetyltransferase and homocitrate synthase (colored in green in Figure 4C) are responsible for producing intermediates from acetyl-CoA for fatty acid biosynthesis, butanoate metabolism and leucine biosynthesis, respectively (Figure S4). In all, the N2 and N3 samples exhibited large capacities for converting pyruvate to pivotal intermediates for carbohydrates, fatty acids and amino acids, which further contribute to specific flavor.

Pyruvate can also be converted to lactic acid in conditions of insufficient oxygen, which likely occurs inside the liquor starter brick. In the NF liquor starter, low numbers of L-lactate dehydrogenase (cytochrome), D-lactate dehydrogenase, D-lactate dehydrogenase (cytochrome) and L-lactate dehydrogenase were detected in the N1 (total RPKM: 80.5) and N4 (165.0) samples, but higher numbers were found in N2 (RPKM: 611.2) and N3 (RPKM: 600.2) (Figures 4B,C and Table S7).

### The citrate cycle and flavor generation

A high total number of citrate cycle genes were found in the N2 sample (Figure 1). However, the number of key enzymes increased in N3 (Figure S5). Most of the top 24 most highly expressed citrate cycle enzymes had definitively higher expression levels in N2 and N3, with 13 enzyme genes having their highest expression levels in N3 and 6 genes in N2 (Figure 4D and Table S8). Meanwhile, isocitrate dehydrogenase (NAD+) (IDH3) and isocitrate dehydrogenase (IDH1) showed comparably high expression levels in N2 and N3. Furthermore, a comparison between the N2 and N3 samples was deeply analyzed, and most of the key enzyme genes were up-regulated in N3. The RPKM and log_2_Ratio (N3/N2) values of the key enzymes, citrate synthase, isocitrate dehydrogenase, 2-oxoglutarate dehydrogenase, dihydrolipoamide succinyl transferase and dihydrolipoamide dehydrogenase, are shown in Table 6. These key enzymes were mainly produced by bacterial and fungal community members such as *Eurotiomycetidae, Bacillales, Trichocomaceae, Firmicutes*, and *E. nidulans*.

**Table 6:** Changes in citrate synthase (EC2.3.3.1), isocitrate dehydrogenase (EC1.1.1.42 and EC1.1.1.41), 2-oxoglutarate dehydrogenase (EC1.2.4.2), dihydrolipoamide succinyl transferase (EC2.3.1.61), and dihydrolipoamide dehydrogenase (EC1.8.1.4), key enzymes in the citrate cycle, in liquor starter samples N3 and N2.

**GeneID**	**Ko_defi**	**Ko_EC**	**N2-RPKM**	**N3-RPKM**	**log_2_ Ratio (N3/N2)**	**Up-Down-Regulation (N3/N2)**	***P*-value**	**FDR**	**Species information**
N3_22492	Citrate synthase	2.3.3.1	0.8	5.5	2.9	Up	1.15E-05	1.71E-05	*Eurotiomycetidae*
N3_26467	Citrate synthase	2.3.3.1	0.0	55.4	15.8	Up	4.44E-16	1.48E-15	*Bacillales*
N3_30325	Citrate synthase	2.3.3.1	0.0	165.1	17.3	Up	4.44E-16	1.92E-15	*Bacillales*
N3_41412	Citrate synthase	2.3.3.1	0.0	130.5	17.0	Up	4.44E-16	1.63E-15	*Trichocomaceae*
N3_20330	Isocitrate dehydrogenase (IDH1)	1.1.1.42	30.4	202.7	2.7	Up	0	0	*Eurotiomycetidae*
N3_26466	Isocitrate dehydrogenase (IDH1)	1.1.1.42	0.0	54.0	15.7	Up	4.44E-16	1.34E-15	*Firmicutes*
N3_30327	isocitrate dehydrogenase (IDH1)	1.1.1.42	0.0	122.5	16.9	Up	4.44E-16	1.46E-15	*Firmicutes*
N3_723	Isocitrate dehydrogenase (IDH1)	1.1.1.42	0.0	52.5	15.7	Up	4.44E-16	1.45E-15	*Trichocomaceae*
N3_6975	Isocitrate dehydrogenase (IDH1)	1.1.1.42	0.0	116.1	16.8	Up	4.44E-16	1.69E-15	*Eurotiomycetidae*
N3_21675	Isocitrate dehydrogenase (NAD+) (IDH3)	1.1.1.41	26.6	127.7	2.3	Up	1.60E-13	4.10E-13	*Trichocomaceae*
N3_36039	Isocitrate dehydrogenase (NAD+) (IDH3)	1.1.1.41	0.0	62.9	15.9	Up	4.44E-16	1.51E-15	*Trichocomaceae*
N3_25689	2-oxoglutarate dehydrogenase E1	1.2.4.2	0.0	131.4	17.0	Up	4.44E-16	1.85E-15	*Eurotiomycetidae*
N3_25690	2-oxoglutarate dehydrogenase E1	1.2.4.2	11.5	89.7	3.0	Up	0	0	*Eurotiomycetidae*
N3_32950	2-oxoglutarate dehydrogenase E1	1.2.4.2	0.3	15.5	5.8	Up	0.000137615	0.000182959	Bacteria
N3_17371	2-oxoglutarate dehydrogenase E2	2.3.1.61	0.0	27.6	14.8	Up	4.44E-16	1.75E-15	*Emericella nidulans*
N3_21757	2-oxoglutarate dehydrogenase E2	2.3.1.61	24.8	156.1	2.7	Up	4.11E-13	1.03E-12	*Proteobacteria*
N3_9942	2-oxoglutarate dehydrogenase E2	2.3.1.61	0.0	53.8	15.7	Up	4.44E-16	1.43E-15	*Emericella nidulans*
N3_32516	Dihydrolipoamide dehydrogenase	1.8.1.4	0.0	35.0	15.1	Up	4.44E-16	1.35E-15	*Bacillales*
N3_33478	Dihydrolipoamide dehydrogenase	1.8.1.4	0.0	64.4	16.0	Up	4.44E-16	1.62E-15	*Bacillales*
N3_12553	Dihydrolipoamide dehydrogenase	1.8.1.4	30.0	307.4	3.4	Up	6.75E-13	1.66E-12	*Trichocomaceae*

## Discussion

This study globally and comprehensively explored the active microbial community and its function in the highly consumed NF liquor starter. A promising number of thermophilic enzymes were also discovered. The results showed that the fungal communities were much more diverse and the enzymes produced by them were more abundant than that of bacteria communities during the process of making the liquor starter, especially during the high temperature period. This interesting finding is complementary to the results of 16S rRNA and ITS sequencing study (Huang et al., unpublished data), which indicated that the diversity and richness of the total bacterial community was much higher than that of the total fungal community. Thus, metatranscriptomics offered an important and excellent platform to actually understand the dynamics of microbial metabolism at the transcript level in liquor starter.

In this study, we discovered diverse and abundant carbohydrate-active enzymes from the NF liquor starter, especially in the N2 and N3 samples, which were characterized by high temperature and an aerobic environment. As mentioned above (Table 2), some thermostable carbohydrate-active enzymes were only detected in special stages, such as endo-1,3-β-D-glucanase and endo-1,5-α-L-arabinanase in N2, rhamnogalacturonanase in N3 and N4, endo-proteases and endo-β-1,3-1,4-glucanase in N2, N3, and N4, and endo-1,4-β-D-xylanase in N4. Thus, this study highlights the benefits of specifically mining for thermostable enzymes from one special stage (N2, N3, or N4) and not just from the mature starter (N4). The liquor starter production system is markedly different from other types of environmental systems, such as the microbes in cow rumens (Hess et al., 2011), wood-feeding termite hindguts (Warnecke et al., 2007), leaf-cutter ant fungal gardens (Aylward et al., 2012), and panda guts (Zhu et al., 2011), which have been well studied using metagenomic and metaproteomic strategies. Relatively high numbers of carbohydrate-active enzymes have been found in these systems by metagenomic technologies (Table S9). However, these metagenomic analyses could not reflect the genes that are actively expressed at any given time and in response to external environmental conditions. Additionally, the microbial communities associated with these various gut systems were dominated by anaerobic bacterial taxa. Notably, industrial-scale lignocellulose degradation has mostly been demonstrated under aerobic conditions (Robinson et al., 2001). By contrast, liquor starter is made in an aerobic environment, and both bacteria and fungi were enriched, with the microbial composition dynamically changing during the production process. The highest number of carbohydrate-active enzymes was found at 62°C and thus potentially offers thermophilic enzymes for lignocellulosic biomass degradation. The enzymatic conversion of polysaccharides in agricultural waste is a promising technology. However, it is still limited by the heterogeneity of the plant cell wall and recalcitrant biomass (Himmel et al., 2007). Previous studies have found that AA9 and AA10 can act synergistically with cellulose, hemicellulose, starch and chitin (Harris et al., 2010; Horn et al., 2012; Lo Leggio et al., 2015; Paspaliari et al., 2015; Kojima et al., 2016) because they have flat substrate-binding surfaces and have an oxidative mechanism to cleave polysaccharide chains in the crystalline context (Vaaje-Kolstad et al., 2010). The AA9 genes in the N3 sample were produced by *T. aurantiacus, Trichocomaceae*, and *Emericella nidulans*. *Thermoascus aurantiacus* is a promising thermophilic fungus for enzyme production and biomass degradation (McClendon et al., 2012). One of the *T. aurantiacus* AA9 enzymes can reduce commercial enzyme loads and is part of a well-understood synergistic system (Rosgaard et al., 2006; Harris et al., 2010). The *Trichocomaceae* family has been reported to have diverse physiological properties and can grow under extreme conditions. Some members of this family have been exploited in biotechnology for the production of enzymes (Houbraken, 2013). The thermotolerant *E. nidulans* (also called *Aspergillus nidulans*) can also utilize a broad spectrum of biomass to produce enzymes with high specific activities (Kango et al., 2003). The present study was the first to identify an AA10 protein from *Bacillales* in liquor starter, which might boost cellulose degradation. These highly expressed AA9 and AA10 members might contribute to the robust degradation capabilities of NF liquor starter, and made themselves potential candidates for industrial application. Based on these results, approximately 60 complete carbohydrate-active enzyme genes, including several AA9 proteins sequences, have been amplified from the cDNA of sample N3. They will be further cloned, expressed and characterized in future work.

Functional profiling and comparative analysis of the 4 NF liquor starter samples showed that oxidative phosphorylation was the most abundant pathway in sample N2, indicating that the microbial community quickly metabolized and released ample energy to drive energy-requiring reactions, as well as producing considerable heat that increased the room temperature to 50°C in 3 days. Among the 20 abundant pathways, most of them were closely related to energy and sugar metabolism, indicating that the microbial community has a great capability to degrade sugars and convert them to important products, such as ethanol. Additionally, butanoate metabolism and propanoate metabolism were also active in the liquor starter samples. Butanoate and propanoate are the most important substrates for ethyl caproate biosynthesis. Ethyl caproate is a key component that affects the flavor and quality of NF liquor (Tao et al., 2014). More interestingly, amino acid metabolism was robust in the NF liquor starter. The metabolisms of 10 amino acids were found to be dominant in the N2 and N3 samples. Aliphatic and branched-chain amino acids are the main pre-substrates for liquor flavor generation (Zhuang, 2007). Thus, these highly expressed genes involved in butanoate, propanoate and amino acid metabolism indicate that the liquor starter has great potential for flavor development.

We further analyzed starch metabolism, glycolysis and ethanol and pyruvate metabolism because they are important for ethanol production and are related to flavor generation. The results showed that the microbial community had a high capability for degrading starch with different functional enzymes throughout the liquor starter production process, especially during the high temperature period. The key enzymes of glycolysis metabolism in the N2 and N3 samples were highly expressed. All the up-regulated genes in the glycolysis pathways were mainly from *Saccharomycetales* and *Mucorales*. *Saccharomycetales* are multifunctional microorganisms that saccharify sugar polymers, improve esterification, contribute to aroma precursors, utilize feedstocks efficiently and affect flavor. *Mucorales* belongs to the Zygomycetes family of filamentous fungi. They are robust fungi that show great promise for ethanol fermentation (Abedinifar et al., 2009) and the production of efficient and diverse carbohydrate-active enzymes (Battaglia et al., 2011). Therefore, the extremely highly expressed key glycolytic enzymes from *Saccharomycetales* and *Mucorales* were important for saccharification and ethanol fermentation. Additionally, more diverse microbial community members, including both fungi and bacteria, mainly contributed to pyruvate and ethanol metabolism at 50 and 62°C. Analysis of the important enzymes of pyruvate metabolism further showed that the NF liquor starter exhibited large capacities for converting pyruvate to intermediates, i.e., acetate and acetyl-CoA, which further contribute to other metabolic functions and specific flavor. The enzymes involved in the conversion of pyruvate to lactic acid were higher in the N2 and N3 samples and reduced in N4 sample. To some extent, this result was consistent with finding in the study by Huang et al. (unpublished data); *Lactobacillus* increased quickly when the liquor starter had incubated for 3 days (N2) and decreased markedly when the temperature reached its maximum of 62°C (N3), and fewer was found in N4. A markedly low level of lactic acid in the mature NF liquor starter is associated with high quality, and such starter can be further used for ethanol fermentation (Li, 2000; Lai, 2007).

The intermediates of the citrate cycle also have important functions in specific flavor generation. Thus, the citrate cycle is essential for many biochemical pathways in the liquor starter microbial community and it is necessary to understand the multiple functions of this cycle in the liquor starter process. The key enzymes of the citrate cycle were produced by both bacterial and fungal community members in the N2 and N3 samples. *Bacillales* species were the dominant bacterial community members when the room temperature increased to 62°C (Huang et al., unpublished data). The other relevant microbial community members were fungi. The high expression levels of the fungal community members also confirmed the high abundance of the active fungal community at the highest temperature point. All of these fungi have been reported to have high capabilities for degrading carbohydrates, fats and amino acids. Therefore, the N2 and N3 samples were more active and would have large capacities for generating energy and producing intermediates for liquor flavor generation. However, liquor flavor development is determined by a much more complex metabolic process; it is determined not only by the microbial community from the liquor starter but also by the Zaopei and pit mud. Thus, the mechanism of liquor flavor generation requires further comprehensive analysis of the microbial communities from the liquor starter, Zaopei and pit mud.

## Conclusions

Chinese liquor starter is produced in a thermophilic and aerobic system. The present study used metatranscriptomics to globally and comprehensively explore the active microbial communities and their functional transcripts in NF liquor starter. The results demonstrated that fungi were the most abundant active community members during the liquor starter production process. The identified abundant pathways, diverse thermophilic carbohydrate-active enzymes, and up-regulated key enzyme genes that are involved in glycolysis, ethanol metabolism, pyruvate metabolism and the citrate cycle at 50 and 62°C implied that the liquor starter is capable of robust saccharification, fermentation and production of flavor-generating agents. A breakthrough occurred during this study regarding the understanding of microbial metabolism and the function of Chinese liquor starter, paving the way for the optimization of liquor production and for the discovery of special and scarce microbial resources and thermophilic enzymes. To obtain encompassing insights into Chinese liquor, which has been produced for several thousand years and involves a complex and dynamic ecosystem, further temporal and spatial studies are needed concerning the microbial communities involved throughout the entire liquor brewing process.

## Author contributions

HZ, YH, KH, DL, HL, YJ, and YF designed the experiment; YH and ZY wrote the main manuscript, performed the experiment and analyzed data; YH, DZ, and HH collected samples and communicated with the liquor factory; YH, ZY, YF, and HZ revised the manuscript. All authors revised and approved the final version of the manuscript.

### Conflict of interest statement

The authors declare that the research was conducted in the absence of any commercial or financial relationships that could be construed as a potential conflict of interest.
