# Supplementary material for: Metatranscriptomics Reveals the Functions and Enzyme Profiles of the Microbial Community in Chinese Nong-Flavor Liquor Starter
Source: Front Microbiol. 2017 Sep 12;8:1747. doi: 10.3389/fmicb.2017.01747 (PMC5600954; doi:10.3389/fmicb.2017.01747)
Supplement: Supplementary file 1 [file DataSheet1.docx]

Supplementary Material

Metatranscriptomics reveals the function and enzyme profiles of microbial communities in Chinese Nong-flavor liquor starter

Yuhong Huang^1,2,3#^, Zhuolin Yi^2,3#^, Yanling Jin^2,3^, Mengjun Huang^2,3^, Kaize He^2,3^, Dayu Liu^1^, Huibo Luo^4^, Dong Zhao^5^, Hui He^6^, Yang Fang^2,3 *^, Hai Zhao^1,2,3*^

^1 Meat-processing Application Key Laboratory of Sichuan Province, College of Pharmacy and Biological Engineering, Chengdu University, Chengdu, China^

^2 Environmental Microbiology Key Laboratory of Sichuan Province, Chengdu Institute of Biology, Chinese Academy of Sciences, Chengdu, China^

^3 Key Laboratory of Environmental and Applied Microbiology, Chinese Academy of Sciences, Chengdu, China^

^4 Liquor Making Bio-Technology & Application of Key Laboratory of Sichuan Province, Bioengineering College, Sichuan University of Science & Engineering, Zigong, China^

^5 Wuliangye Group, Yibin, China^

^6 Department of liquor making engineering, Moutai College, Renhuai, China^

*** Correspondence:** Hai Zhao (email: zhaohai@cib.ac.cn) and Yang Fang (email: fangyang@cib.ac.cn), Environmental Microbiology Key Laboratory, Chengdu Institute of Biology, CAS, No.9 Section 4, Renmin Nan Road, Chengdu, 610041, Sichuan, P.R. China. Phone: +86 28 82890725; Fax: +86 28 82890733;

^#^These authors contributed equally to this work.

# Supplementary Figures


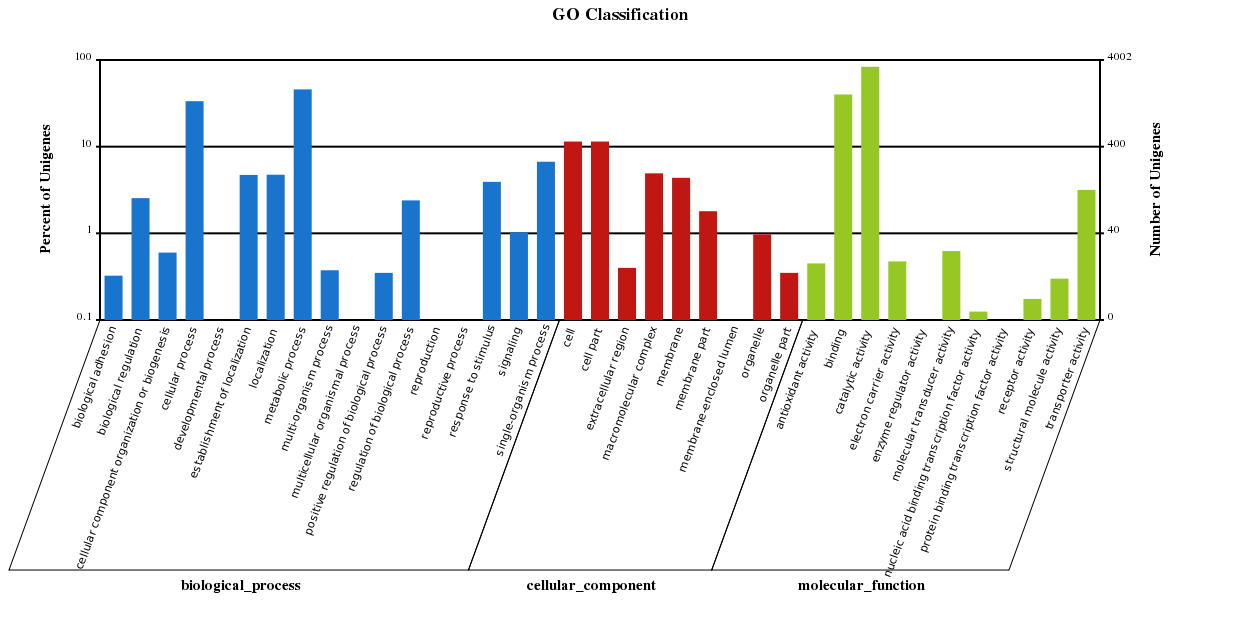


**Figure S1** Gene ontology (GO) classification of the Nong-flavor liquor starter samples. The Unigenes of Nong-flavor liquor starter samples N1-N4 were pooled together. The GO classification was achieved using WEGO. N1 was sampled at the beginning of liquor starter production; N2 was sampled after 3 days of liquor starter fermentation; N3 was sampled after 9 days of liquor starter fermentation; N4 was the mature liquor starter. The temperatures of N1, N2, N3 and N4 were 30, 50, 62 and 25 °C, respectively.

**
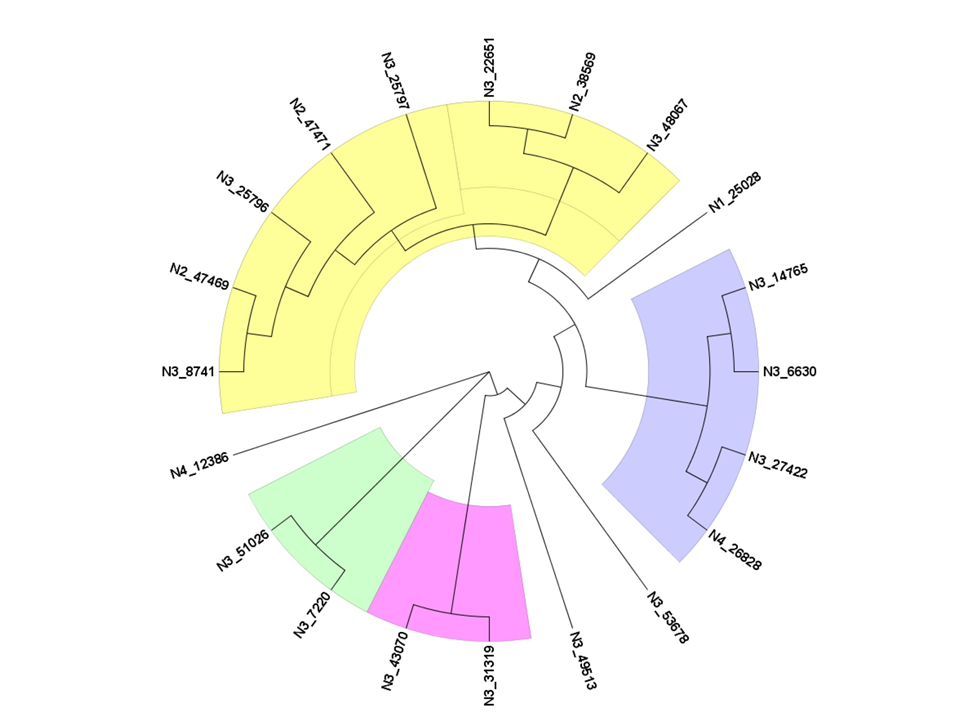
**

**Figure S2** Phylogenetic tree based on the protein sequences of the AA9 and AA10 families in Nong-flavor metatranscriptome. The gene information can be found in Table 2. The phylogenetic tree was constructed by the neighbor-joining method using CLUSTAL X and Figtree. The levels of bootstrap support are indicated at the nodes.


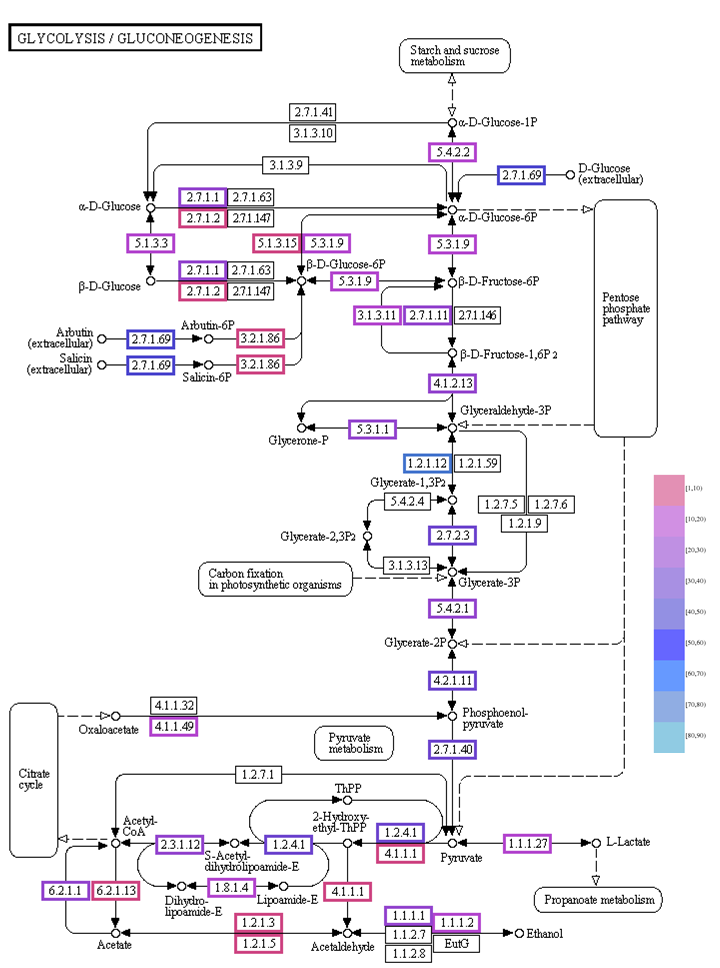


**Figure S3** Expression patterns of glycolysis and ethanol metabolism pathway related transcripts (N2). Expression variations were indicated in color boxes according to the color direction (right).

**
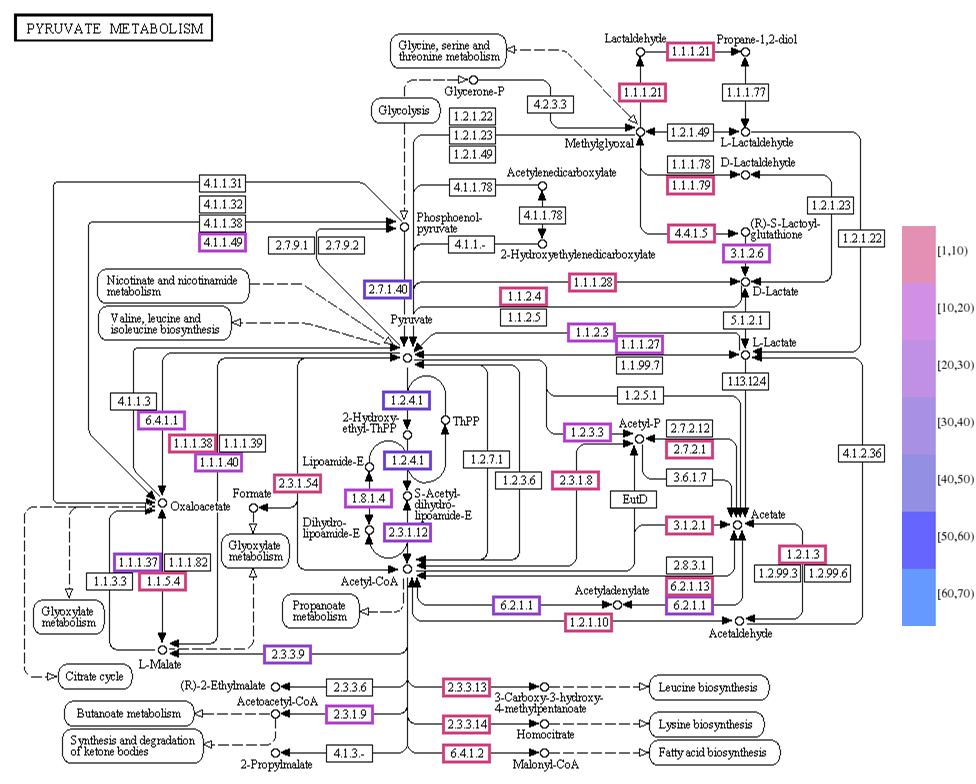
**

**Figure S4** Expression patterns of pyruvate metabolism (N2). Expression variations was indicated in color boxes according to the color direction (right)


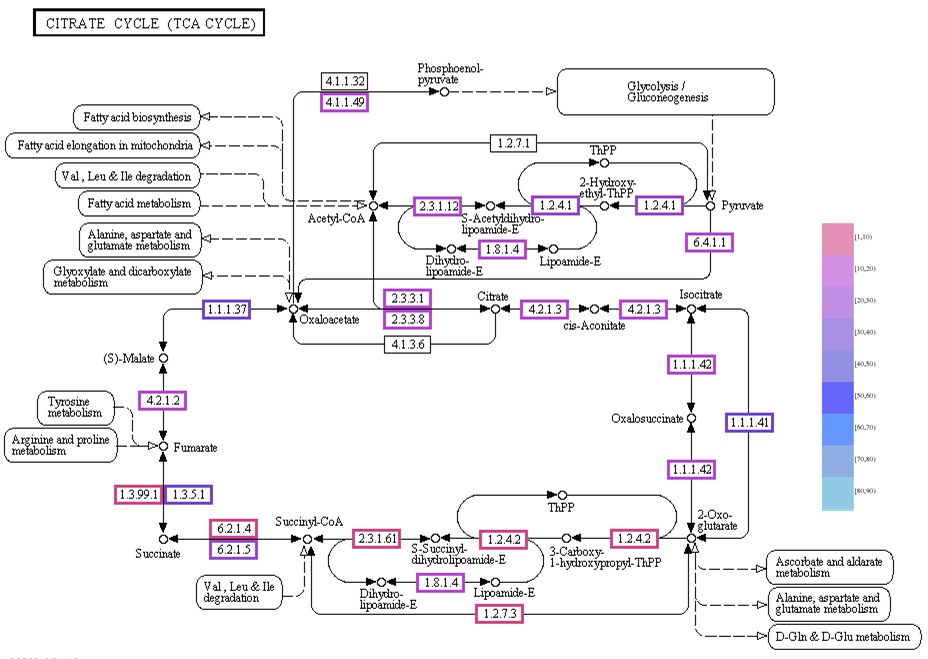


**Figure S5** Expression patterns of TCA cycle related transcripts (N3). Expression variations was indicated in color boxes according to the color direction (right)

# Supplementary Tables

**Table S1** Raw sequencing data and clean data statistic of the liquor starter metatranscriptome. N1 was sampled at the beginning of liquor starter production; N2 was sampled after 3 days of liquor starter fermentation; N3 was sampled after 9 days of liquor starter fermentation; N4 was the mature liquor starter. The temperatures of N1, N2, N3 and N4 were 30, 50, 62 and 25 °C, respectively.

| Sample ID | Insert size (bp) | Sequence type (bp) | Raw reads (M) | High quality reads (M) | Reads remove host | Clean data rate (%) |
| --- | --- | --- | --- | --- | --- | --- |
| N1 | 180 | 100 | 5645 | 5000 | 0.00 | 89 |
| N2 | 180 | 100 | 5492 | 5000 | 0.00 | 91 |
| N3 | 180 | 100 | 5434 | 5000 | 0.00 | 92 |
| N4 | 180 | 100 | 5741 | 5000 | 0.00 | 88 |

**Table S2** Assembly quantity statistics of the liquor starter metatranscriptome. N1 was sampled at the beginning of liquor starter production; N2 was sampled after 3 days of liquor starter fermentation; N3 was sampled after 9 days of liquor starter fermentation; N4 was the mature liquor starter. The temperatures of N1, N2, N3 and N4 were 30, 50, 62 and 25 °C, respectively.

| Sample ID | Contignum | Contiglen (bp) | N50  (bp) | N90  (bp) | Max  (bp) | Min  (bp) | Map to own contigs | | |
| --- | --- | --- | --- | --- | --- | --- | --- | --- | --- |
|  |  |  |  |  |  |  | PE | SE | % |
| N1 | 102,187 | 40,921,929 | 404 | 220 | 13,340 | 201 | 12,978,818 | 7,661,487 | 67.24 |
| N2 | 133,815 | 62,053,491 | 538 | 232 | 17,819 | 201 | 5,004,972 | 20,539,624 | 61.10 |
| N3 | 101,954 | 69,953,277 | 1084 | 275 | 12,496 | 201 | 4,413,397 | 13,979,355 | 45.61 |
| N4 | 59,204 | 39,669,651 | 1105 | 265 | 12,933 | 201 | 6,244,266 | 23,022,279 | 71.02 |

**Table S3** Gene prediction statistic of the liquor starter metatranscriptome.

| Sample ID | ORFs | Total length | Avg.length |
| --- | --- | --- | --- |
| N1 | 25,387 | 15,624,672 | 615.46 |
| N2 | 58,884 | 36,223,758 | 615.17 |
| N3 | 56,927 | 45,278,895 | 795.39 |
| N4 | 28,618 | 23,048,523 | 805.39 |

**Table S4** Functional annotation results of liquor starter based on CAZy (Carbohydrate-Active Enzyme Database)

| Sample ID | GHs | GTs | PLs | CEs | CBMs |
| --- | --- | --- | --- | --- | --- |
| N1 | 178 | 279 | 0 | 25 | 36 |
| N2 | 301 | 344 | 3 | 81 | 48 |
| N3 | 478 | 397 | 0 | 57 | 64 |
| N4 | 237 | 161 | 1 | 22 | 24 |

**Table S5** The changes of key enzymes hexokinase (EC2.7.1.1), 6-phosphofructokinase (EC2.7.1.11) and pyruvate kinase (EC2.7.1.40) in glycolysis pathway between liquor starter sample N2 and N1

| geneID | Ko_name | Ko_defi | Ko_EC | N1-RPKM | N2-RPKM | log2 Ratio(N2/N1) | Up-Down-Regulation(N2/N1) | P-value | FDR | Species information |
| --- | --- | --- | --- | --- | --- | --- | --- | --- | --- | --- |
| N2_28425 | HK | hexokinase | 2.7.1.1 | 0.0 | 8.0 | 13.0 | Up | 0.0258 | 0.0351 | *Saccharomycetales* |
| N2_36044 | HK | hexokinase | 2.7.1.1 | 0.0 | 27.2 | 14.7 | Up | 0.0000 | 0.0000 | *Mucorales* |
| N2_773 | HK | hexokinase | 2.7.1.1 | 0.0 | 66.2 | 16.0 | Up | 0.0000 | 0.0000 | *Mucorales* |
| N2_38063 | HK | hexokinase | 2.7.1.1 | 0.0 | 16.3 | 14.0 | Up | 0.0003 | 0.0006 | *Saccharomycetales* |
| N2_51593 | HK | hexokinase | 2.7.1.1 | 0.0 | 53.7 | 15.7 | Up | 0.0000 | 0.0000 | *Mucorales* |
| N2_56852 | HK | hexokinase | 2.7.1.1 | 0.0 | 2.5 | 11.3 | Up | 0.0832 | 0.1039 | *Mucorales* |
| N2_56853 | HK | hexokinase | 2.7.1.1 | 0.0 | 22.7 | 14.5 | Up | 0.0000 | 0.0000 | *Mucorales* |
| N2_58233 | HK | hexokinase | 2.7.1.1 | 0.0 | 24.3 | 14.6 | Up | 0.0000 | 0.0000 | *Mucorales* |
| N2_18504 | PFK. pfk | 6-phosphofructokinase | 2.7.1.11 | 0.0 | 7.7 | 12.9 | Up | 0.0094 | 0.0137 | *Saccharomycetales* |
| N2_25659 | PFK. pfk | 6-phosphofructokinase | 2.7.1.11 | 0.0 | 13.7 | 13.7 | Up | 0.0094 | 0.0137 | None |
| N2_32202 | PFK. pfk | 6-phosphofructokinase | 2.7.1.11 | 0.0 | 4.9 | 12.2 | Up | 0.2684 | 0.3080 | *Saccharomycetales* |
| N2_37013 | PFK. pfk | 6-phosphofructokinase | 2.7.1.11 | 0.0 | 11.0 | 13.4 | Up | 0.0041 | 0.0063 | None |
| N2_39843 | PFK. pfk | 6-phosphofructokinase | 2.7.1.11 | 0.0 | 14.0 | 13.8 | Up | 0.0009 | 0.0015 | *Mucorales* |
| N2_53798 | PFK. pfk | 6-phosphofructokinase | 2.7.1.11 | 0.0 | 21.5 | 14.4 | Up | 0.0000 | 0.0000 | None |
| N2_7646 | PFK. pfk | 6-phosphofructokinase | 2.7.1.11 | 0.9 | 64.4 | 6.2 | Up | 0.0000 | 0.0000 | *Mucorales* |
| N2_10069 | PFK. pfk | 6-phosphofructokinase | 2.7.1.11 | 0.0 | 112.9 | 16.8 | Up | 0.0000 | 0.0000 | *Mucorales* |
| N2_10628 | PFK. pfk | 6-phosphofructokinase | 2.7.1.11 | 0.0 | 38.7 | 15.2 | Up | 0.0000 | 0.0000 | *Mucorales* |
| N2_10629 | PFK. pfk | 6-phosphofructokinase | 2.7.1.11 | 0.0 | 37.1 | 15.2 | Up | 0.0000 | 0.0000 | *Mucorales* |
| N2_23271 | PK. pyk | pyruvate kinase | 2.7.1.40 | 0.0 | 13.0 | 13.7 | Up | 0.0029 | 0.0046 | *Saccharomycetales* |
| N2_33136 | PK. pyk | pyruvate kinase | 2.7.1.40 | 0.0 | 1.3 | 10.4 | Up | 0.7327 | 0.7778 | None |
| N2_37799 | PK. pyk | pyruvate kinase | 2.7.1.40 | 0.0 | 30.8 | 14.9 | Up | 0.0000 | 0.0000 | None |
| N2_41200 | PK. pyk | pyruvate kinase | 2.7.1.40 | 2.2 | 31.8 | 3.9 | Up | 0.0000 | 0.0000 | None |
| N2_44786 | PK. pyk | pyruvate kinase | 2.7.1.40 | 0.0 | 1.5 | 10.6 | Up | 0.8662 | 0.8894 | *Mucorales* |
| N2_51260 | PK. pyk | pyruvate kinase | 2.7.1.40 | 0.0 | 32.5 | 15.0 | Up | 0.0000 | 0.0000 | *Mucorales* |
| N2_52199 | PK. pyk | pyruvate kinase | 2.7.1.40 | 0.0 | 16.1 | 14.0 | Up | 0.0001 | 0.0002 | None |
| N2_52742 | PK. pyk | pyruvate kinase | 2.7.1.40 | 0.0 | 8.9 | 13.1 | Up | 0.0000 | 0.0000 | *Mucorales* |
| N2_5058 | PK. pyk | pyruvate kinase | 2.7.1.40 | 0.0 | 13.7 | 13.7 | Up | 0.0000 | 0.0000 | None |
| N2_5062 | PK. pyk | pyruvate kinase | 2.7.1.40 | 2.8 | 34.1 | 3.6 | Up | 0.0000 | 0.0000 | None |
| N2_5063 | PK. pyk | pyruvate kinase | 2.7.1.40 | 1.1 | 193.8 | 7.4 | Up | 0.0000 | 0.0000 | *Rhizophydiales* |
| N2_7026 | PK. pyk | pyruvate kinase | 2.7.1.40 | 1.0 | 134.1 | 7.0 | Up | 0.0000 | 0.0000 | *Mucorales* |
| N2_8325 | PK. pyk | pyruvate kinase | 2.7.1.40 | 0.0 | 37.6 | 15.2 | Up | 0.0000 | 0.0000 | *Mucorales* |
| N2_8327 | PK. pyk | pyruvate kinase | 2.7.1.40 | 3.1 | 37.2 | 3.6 | Up | 0.0000 | 0.0000 | *Mucorales* |
| N2_15023 | PK. pyk | pyruvate kinase | 2.7.1.40 | 0.0 | 17.2 | 14.1 | Up | 0.0015 | 0.0024 | *Saccharomycetales* |
| N2_15518 | PK. pyk | pyruvate kinase | 2.7.1.40 | 0.0 | 14.2 | 13.8 | Up | 0.0021 | 0.0033 | *Mucorales* |
|  |  |  |  |  |  |  |  |  |  |  |

**Table S6** Relative high expression of genes related to Pyruvate metabolism in liquor starter samples (N1, N2, N3 and N4).

| Gene function | EC number | RPKM values | | | |  |
| --- | --- | --- | --- | --- | --- | --- |
|  |  | N1 | N2 | N3 | N4 | Total |
| lactoylglutathione lyase | 4.4.1.5 | 293.4 | 734.8 | 745.6 | 108.4 | 1882.1 |
| acetyl-CoA C-acetyltransferase | 2.3.1.9 | 175.9 | 396.2 | 455.7 | 181.3 | 1209.1 |
| hydroxyacylglutathione hydrolase | 3.1.2.6 | 256.6 | 293.7 | 437.0 | 49.1 | 1036.5 |
| acetyl-CoA carboxylase (ACAC) | 6.4.1.2 | 48.7 | 450.8 | 213.3 | 169.3 | 882.1 |
| D-lactate dehydrogenase (cytochrome) | 1.1.2.4 | 62.1 | 168.2 | 439.6 | 97.8 | 767.7 |
| malate dehydrogenase | 1.1.1.38 | 82.3 | 368.4 | 219.2 | 32.4 | 702.2 |
| malate synthase | 2.3.3.9 | 0.0 | 105.9 | 290.3 | 265.2 | 661.4 |
| acetate kinase | 2.7.2.1 | 1.3 | 154.8 | 16.5 | 393.6 | 566.2 |
| acetyl-CoA hydrolase | 3.1.2.1 | 2.1 | 142.3 | 291.9 | 105.9 | 542.2 |
| 2-isopropylmalate synthase | 2.3.3.13 | 43.7 | 116.4 | 149.1 | 160.6 | 469.8 |
| pyruvate oxidase | 1.2.3.3 | 2.9 | 374.6 | 38.5 | 37.9 | 453.9 |
| phosphoenolpyruvate carboxylase | 4.1.1.31 | 419.2 | 1.7 | 0.0 | 0.0 | 420.8 |
| L-lactate dehydrogenase (cytochrome) | 1.1.2.3 | 2.5 | 211.7 | 136.2 | 42.2 | 392.7 |
| homocitrate synthase | 2.3.3.14 | 0.0 | 86.2 | 155.5 | 121.9 | 363.7 |
| acetyl-CoA carboxylase carboxyl transferase (accA) | 6.4.1.2 | 21.2 | 142.1 | 57.7 | 6.0 | 227.0 |
| D-lactate dehydrogenase (ldhA) | 1.1.1.28 | 0.0 | 165.9 | 2.2 | 11.0 | 179.2 |
| formate C-acetyltransferase | 2.3.1.54 | 2.9 | 130.8 | 0.0 | 41.7 | 175.4 |
| phosphate acetyltransferase | 2.3.1.8 | 0.0 | 144.1 | 2.6 | 16.3 | 163.0 |
| pyruvate, water dikinase | 2.7.9.2 | 0.0 | 0.0 | 98.5 | 1.9 | 100.4 |

**Table S7** The changes of L-lactate dehydrogenase and D-lactate dehydrogenase in glycolysis and pyruvate metabolism among four liquor starter samples.

| Metabolism | Gene function | EC number | RPKM values (total) | | | |
| --- | --- | --- | --- | --- | --- | --- |
|  |  |  | N1 (80.5) | N2 (661.2) | N3 (600.2) | N4 (165.0) |
| Glycolysis | L-lactate dehydrogenase | 1.1.1.27 | 15.9 | 115.4 | 22.2 | 14.0 |
| Pyruvate metabolism | D-lactate dehydrogenase (cytochrome) | 1.1.2.4 | 62.1 | 168.2 | 439.6 | 97.8 |
|  | L-lactate dehydrogenase (cytochrome) | 1.1.2.3 | 2.5 | 211.7 | 136.2 | 42.2 |
|  | D-lactate dehydrogenase | 1.1.1.28 | 0.0 | 165.9 | 2.2 | 11.0 |

**Table S8** Relative high expression of genes related to citrate cycle in liquor starter samples (N1, N2, N3 and N4).

| Gene function | Ko_EC | RPKM values | | | | |
| --- | --- | --- | --- | --- | --- | --- |
|  |  | N1 | N2 | N3 | N4 | Total |
| malate dehydrogenase (MDH2) | 1.1.1.37 | 444.6 | 508.0 | 849.5 | 158.6 | 1960.6 |
| aconitate hydratase 1 | 4.2.1.3 | 416.4 | 979.4 | 385.0 | 26.1 | 1806.9 |
| ATP citrate (pro-S)-lyase | 2.3.3.8 | 133.9 | 538.8 | 809.8 | 277.6 | 1760.1 |
| 2-oxoglutarate dehydrogenase E1 | 1.2.4.2 | 286.0 | 403.5 | 237.8 | 764.3 | 1691.7 |
| isocitrate dehydrogenase (NAD+) (IDH3) | 1.1.1.41 | 236.0 | 412.6 | 421.2 | 362.5 | 1432.3 |
| succinate dehydrogenase (ubiquinone) (SDHB) | 1.3.5.1 | 387.0 | 390.7 | 375.8 | 206.1 | 1359.7 |
| succinate dehydrogenase (ubiquinone) (SDHA) | 1.3.5.1 | 193.8 | 194.2 | 464.5 | 157.1 | 1009.6 |
| 2-oxoglutarate dehydrogenase E2 | 2.3.1.61 | 295.4 | 355.0 | 238.2 | 114.3 | 1002.9 |
| citrate synthase | 2.3.3.1 | 473.9 | 62.3 | 337.9 | 39.8 | 913.9 |
| succinate dehydrogenase (ubiquinone) (SDHC) | 1.3.5.1 | 194.7 | 242.8 | 287.4 | 129.6 | 854.5 |
| isocitrate dehydrogenase (IDH1) | 1.1.1.42 | 151.1 | 221.3 | 224.0 | 72.7 | 669.1 |
| pyruvate carboxylase | 6.4.1.1 | 2.8 | 265.9 | 185.5 | 106.2 | 560.5 |
| fumarate hydratase, class II | 4.2.1.2 | 49.6 | 161.3 | 300.7 | 47.7 | 559.3 |
| succinate dehydrogenase (ubiquinone) (SDHD) | 1.3.5.1 | 0.0 | 243.2 | 144.9 | 33.2 | 421.3 |
| succinyl-CoA synthetase (LSC1) | 6.2.1.4 | 48.6 | 71.2 | 144.1 | 74.3 | 338.2 |
| succinyl-CoA synthetase (LSC2) | 6.2.1.4 | 70.3 | 137.7 | 92.9 | 17.6 | 318.5 |
| 2-oxoglutarate ferredoxin oxidoreductase (korB) | 1.2.7.3 | 0.0 | 0.0 | 229.0 | 13.5 | 242.5 |
| malate dehydrogenase (mdh) | 1.1.1.37 | 0.0 | 10.5 | 181.3 | 0.0 | 191.8 |
| malate dehydrogenase (MDH1) | 1.1.1.37 | 164.2 | 0.6 | 0.0 | 0.0 | 164.8 |
| 2-oxoglutarate ferredoxin oxidoreductase (korA) | 1.2.7.3 | 0.0 | 0.0 | 156.3 | 5.5 | 161.8 |
| succinyl-CoA synthetase (sucD) | 6.2.1.5 | 0.0 | 0.0 | 114.9 | 6.1 | 121.0 |
| fumarate hydratase, class I | 4.2.1.2 | 0.0 | 0.0 | 115.5 | 3.6 | 119.1 |
| succinate dehydrogenase (sdhB) | 1.3.99.1 | 0.0 | 0.0 | 76.4 | 2.4 | 78.8 |
| succinate dehydrogenase (sdhA) | 1.3.99.1 | 0.0 | 0.0 | 76.0 | 0.7 | 76.7 |

**Table S9** Comparison of predicted carbohydrate-active enzymes in five cellulosic systems: panda gut, leaf-cutter ant fungus gardens, wood-feeding termite hindgut, cow rumen and Nong-flavor liquor starter

| System | Method | Carbohydrate-active enzymes | Environment | Main community | Reference |
| --- | --- | --- | --- | --- | --- |
| panda gut | metagenome | 448 | Anaerobic, common temperature | Bacteria | ([Zhu et al., 2011](#_ENREF_41)) |
| leaf-cutter ant fungus gardens | metaproteome | 176–566 | Anaerobic, common temperature | Bacteria | ([Aylward et al., 2012](#_ENREF_5)) |
| wood-feeding termite hindgut | metagenome | 1267 | Anaerobic, common temperature | Bacteria | ([Warnecke et al., 2007](#_ENREF_37)) |
| cow rumen | metagenome | 27755 | Anaerobic, common temperature | Bacteria | ([Hess et al., 2011](#_ENREF_15)) |
| Nong-flavor liquor starter | metatranscriptome | 996-2736 | Aerobic, 62 °C, low humid | Fungi and bacteria | This research |
